# Safety and Efficacy of Midface Augmentation Using Bio-Oss Bone Powder and Bio-Gide Collagen Membrane in Asians

**DOI:** 10.3390/jcm12030959

**Published:** 2023-01-26

**Authors:** Jia-Yu Zhang, Ke Liu, Ruo-Xi Liu, Bao-Hua Xu

**Affiliations:** 1 China–Japan Friendship Hospital (Institute of Clinical Medical Sciences), Chinese Academy of Medical Sciences & Peking Union Medical College, Beijing 100029, China; 2 China–Japan Friendship Hospital Stomatology Center, Beijing 100013, China

**Keywords:** midface augmentation, nasolabial fold, guided bone regeneration, biomaterial

## Abstract

(1) Background: Asians tend to have a regressive midface. Midface augmentation is an effective treatment, and various materials have been used as fillers for this purpose. Bio-Oss bone powder has a strong positive effect on promoting new bone regeneration, and has been used in the dental field for over 30 years. However, it has not been used and reported as a filler in midface augmentation. (2) Objective: To evaluate the safety and efficacy of midface augmentation using Bio-Oss bone powder in treating midface retrusion and resulting nasolabial folds, and to develop a predictive model for patient satisfaction. (3) Methods: 85 patients underwent midface augmentation through an intraoral approach with Bio-Oss. Treatment efficacy was assessed by blinded investigators. The data on safety were collected from patient interviews at each follow-up visit. A questionnaire was used for investigating patient satisfaction. The influencing factors of satisfaction were analyzed by univariate and multivariate analysis. A nomogram to predict the risk of dissatisfaction was built based on significant factors with R software. Results: Compared to baseline, there was a significant improvement (*p* < 0.001) in Wrinkle Severity (4) Rating Scale scores at week 24, with a mean decrease of 0.52 ± 0.57. The aesthetic improvement rate evaluated by the Global Aesthetic Improvement Scale was 92.9%. Four mild treatment-related adverse events were noted. The majority of patients were satisfied overall. A nomogram with good prediction performance was plotted. (5) Conclusions: This new procedure yielded safe and satisfactory aesthetic results. A nomogram with good test performance and discriminative ability was established for predicting patient satisfaction.

## 1. Introduction

A significant proportion of Asians have flat or sunken midface features. These features tend to be accompanied by some negative aesthetic issues, such as a narrow nasolabial angle, deep nasolabial folds, and a depressed nasal base, which give these patients a gloomy, tired, depressed, and aged appearance. It may be a consequence of maxillary recession or partial hypoplasia of the maxilla around the pyriform aperture [1]. Although a severe retrusion of maxilla requires a large graft for augmentation or a Le Fort osteotomy, mild retrusion around the pyriform aperture can be improved by a simple filling procedure. The existing fillers include autogenous bone [2], autologous cartilage [3], autologous fat [4], autologous fibroblast [5], collagen [6], hyaluronic acid [7], calcium hydroxylapatite [8], poly-L-lactic acid [9], expanded polytetrafluoroethylene [10], Medpor [11], etc.

Deproteinized natural bovine mineral bone powder (Bio-Oss) is a carbonate apatite crystal extracted from bovine bone. After special treatment, proteins and other organic components are removed, making its structure almost identical to human bone [12]. Bio-Oss thus has excellent biocompatibility, good bone conductivity, low biodegradation rate, and minimal tissue reaction [13]. The unique structure and large surface area of Bio-Oss provide an ideal frame structure for the regeneration of new bone [14]. Moreover, with guided new bone regeneration, Bio-Oss will undergo slow biodegradation [12]. At present, Bio-Oss bone powder is the most widely used bone substitute, and its osteogenic effect has been recognized. Bio-Gide is a newly developed bioabsorbable collagen membrane that does not need to be removed by secondary surgery [15]. It is made of non-cross-linked porcine Type I and III collagens, and has a bilayered structure [16]. In united applications, Bio-Oss is used to guide bone regeneration and support subjacent space, while Bio-Gide is used to maintain the overall shape and exclude unwanted epithelial and connective tissue in growth [17]. In this study, we pioneered the use of Bio-Oss combined with Bio-Gide for midface augmentation.

With the aim of evaluating the safety and effectiveness of this new technique, we recruited 85 patients with midface depressions and resulting nasolabial folds to apply this new surgical technique, and collected perioperation information. In addition, we analyzed patients’ satisfaction and constructed a predictive model. To our knowledge, this is the first study on midface augmentation using Bio-Oss bone powder and Bio-Gide collagen membrane.

## 2. Methods and Materials

### 2.1. Subject Selection

Between May 2019 and June 2022, 85 patients underwent midface augmentation with Bio-Oss bone powder through an intraoral approach. The motivation was the patients’ desire to improve midface depression, the resulting nasolabial folds, and their overall facial appearance. Contraindications included obvious anxiety state; pregnant or nursing women; history of hypersensitivity or anaphylactic shock; anticoagulant use within 4 weeks before screening, or history of the hemorrhagic disease; active disease on or near the nasolabial folds; occurrence of nasolabial folds other than the bone retrusion type [18]. Five potential participants were excluded due to contraindications.

### 2.2. Study Design

Each patient was asked to fill out a questionnaire before the procedure, which included demographic information and some additional information of interest to the investigator. Written informed consent was obtained from all patients before enrolment in the study. Each patient received only one midface augmentation treatment within 24 weeks after the screening visit, with no additional treatment. All operations were performed by one surgeon. Follow-up visits were at 4, 12, and 24 weeks after initial treatment, and all patients were photographed at baseline and all follow-up visits. The same photography procedure was used for all photographs. All shooting-related personnel were trained in the same manner, and procedures and equipment were customized to ensure the consistency of all photography files. In order to assess patient satisfaction and related information after surgery, patients were asked to complete another questionnaire at the week 24 follow-up visit. For the patients who failed to come for offline review on time, we followed up by email. Three participants were lost during the follow-up. Adverse events were recorded throughout. This study was approved by the Institutional Review Boards of our hospital (2020-91-K55).

### 2.3. Operation Technique

**Figure 1:**
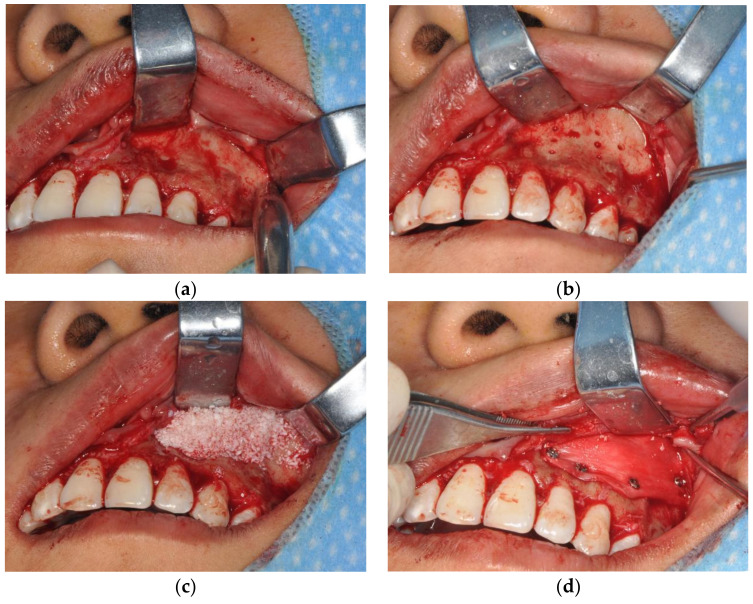
(**a**) The maxilla was exposed; (**b**) 4–8 bleeding holes were prepared with a dental drill in the filling area; (**c**) Bio-Oss bone powder was added to the filling area; (**d**) Bio-Gide collagen membrane was laid on the surface and fixed with titanium screws.

Before the surgery, we listened carefully to the patient’s requests and explained to them the risks and possible outcomes of the procedure. The procedures were performed under local anesthesia with articaine. The range of anesthesia is on both sides of the maxillary first molar or maxillary second molar, up to the root of the nasolabial fold. The dosage was adjusted according to intraoperative patient feedback. We performed an internal beveled incision of the gingiva on the buccal side. The incision is usually up to the top of the alveolar crest, on both sides of the maxillary first molar, and to a subperiosteal depth. Precise anatomical extent and target area of filling were individualized. We then flipped the mucoperiosteal flap and exposed the maxilla (Figure 1a). On each side of the filling area, 4–8 bleeding holes were prepared with a dental drill to facilitate subsequent bone regeneration (Figure 1b). According to the preoperative evaluation, we used different amounts of Bio-Oss bone powder for augmentation to suit the specific depression of each patient. Based on the biodegradability of the Bio-Oss bone powder and previous experience, a 10% overfill was performed (Figure 1c). After the Bio-Oss bone powder was laid on the maxilla, we covered the surface with the Bio-Gide collagen membrane and fixed it with titanium screws (Figure 1d). The filling position was confirmed and adjusted by CT before and after placing the drainage strips. The surgical site was washed with normal saline. Finally, we sutured oral mucosa with 4–0 absorbable sutures.

Perioperative management includes continuous antibiotic application (tinidazole + cephalosporins) for 5 days, revisit for 3 consecutive days after the operation, and instructions for patients to avoid compression of the surgical site and large positive and negative pressure in the oral cavity for 1 week following the operation. The drainage strips were removed on postoperative day 3, and the titanium screws were usually removed 6 months after the operation.

### 2.4. Assessments

#### 2.4.1. Efficacy Assessments

The Wrinkle Severity Rating Scale (WSRS) is an easy, valid, and reliable tool for the quantitative assessment of nasolabial folds, with good intra- and interobserver consistency [19]. By comparing with the standard photographs and descriptions of the WSRS, a classification of 1 to 5 levels can be obtained: absent—1, mild—2, moderate—3, severe—4, and extreme—5 (Supplementary Table S1). The Global Aesthetic Improvement Scale (GAIS) is a five-level scale that rates overall cosmetic improvement by comparing the appearance before and after the treatment [20].

Treatment efficacy was assessed using the WSRS and GAIS [20]. After the last follow-up visit at week 24, evaluators compared each patient’s photographs at each stage to standardized photographs to evaluate the WSRS score. Surgical efficacy was assessed by analyzing the change in WSRS scores for each patient at each stage. The GAIS was used to assess the perception of cosmetic efficacy by blinded evaluators. All background information of the photographs was hidden from the researchers during the evaluation. The primary validity assessment was performed at 24 weeks and the secondary validity assessment at 0, 4, and 12 weeks.

#### 2.4.2. Safety Assessments

The patient’s medical history, medication, and related treatment were evaluated during screening, and a physical examination was performed. Adverse events were defined as undesirable and unexpected events that developed after the treatment. They were evaluated from patient interviews at each follow-up visit.

#### 2.4.3. Satisfaction Assessments

FACE-Q is a new patient-reported assessment tool consisting of more than 40 independently functioning checklists and scales, and is used to evaluate concepts and symptoms important to facial cosmetic patients [21]. A FACE-Q of expectations with scores ranging from 0 to 100 was applied in our study, of which higher scores reflect higher (more unrealistic) expectations.

The patient’s satisfaction was obtained after they were shown photographs at week 24; the levels of satisfaction were categorized into overall satisfied and dissatisfied. For dissatisfied patients, we asked for detailed reasons. Those patients who had failed to make it to the outpatient department were questioned by mail (Supplementary Table S2). In order to screen for factors related to patient satisfaction and develop a predictive model, the following data were collected: gender, age, BMI, ABO blood type, personality, motivation, main purpose, history of periodontal disease, FACE-Q score of expectations [22], systolic blood pressure, diastolic blood pressure, blood glucose, heart rate, operative time, amount of Bio-Oss bone powder, number of titanium screws, size of Bio-Gide collagen membrane, simultaneous orthodontic treatment or not, daily follow-up for 3 days or not. The information on age, gender, motivation, medical history, etc., was obtained from the medical records of the consultation and examination. Information such as the amount of Bio-Oss bone powder was obtained from surgical records and intraoperative photographs.

### 2.5. Statistical Analysis

Continuous variables are shown as the mean and standard deviation (SD), and categorical variables are shown as numbers and proportions. Kolmogorov–Smirnov tests were used to test the normality of continuous variables, and *t*-tests were performed to compare the changes at each follow-up visit from baseline. Categorical variables were analyzed using the x^2^ test and Fisher’s exact test. *p* < 0.05 was considered statistically significant. Factors with *p* < 0.1 in the univariate analysis were incorporated into a multivariate logistic regression analysis to identify the independent influencing factors for satisfaction. The strength of the association was described with odds ratios (ORs) and 95% confidence intervals (CIs). Based on the significant factors, a nomogram was plotted using the regression modeling strategies (rms) program in R software version 3.5.0. In the presence of missing data, valid variables were replaced by baseline data. The SPSS 25.0 software (IBM, Armonk, NY, USA) was used for all statistical analyses.

## 3. Results

### 3.1. Patient Characteristics and Demographics

A total of 85 Asian patients were included in this study. Most of the patients were female (83/85, 97.6%), with a mean age of 27.2 ± 4.2 years. The mean operative time was 101.4 ± 10.3 min. The average filling volume of Bio-Oss bone powder was 2.2 ± 0.5 g.

### 3.2. Efficacy

**Figure 2:**
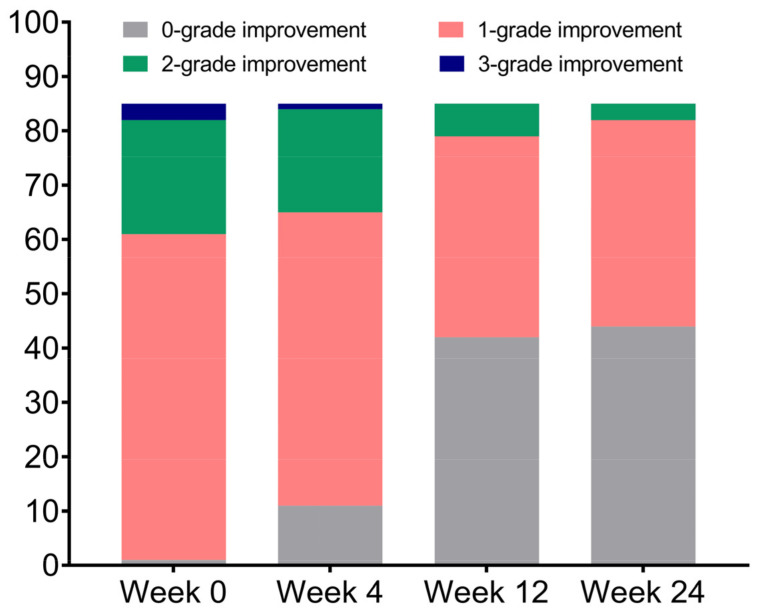
The proportion of WSRS score improvements at week 0, 4, 12, and 24 from baseline. Abbreviations: WSRS = Wrinkle Severity Rating Scale.

**Figure 3:**
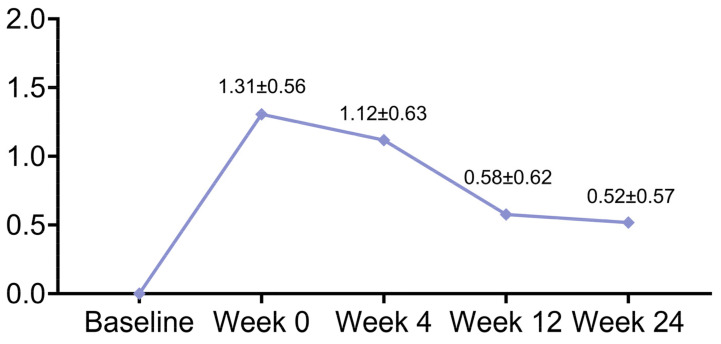
The mean improvement in WSRS scores from baseline at each follow-up visit. Values are the mean ± SD unless otherwise noted. Abbreviations: WSRS = Wrinkle Severity Rating Scale.

At week 24, the WSRS scores improved by 2 grades in 3 patients and by 1 grade in 38 patients (Figure 2). The WSRS scores showed significant improvement (*p* < 0.001) at each follow-up time point compared to baseline (Table 1). The average WSRS score improvements from baseline were 1.31 ± 0.56, 1.12 ± 0.63, 0.58 ± 0.62, and 0.52 ± 0.57 at 0, 4, 12, and 24 weeks, respectively (Figure 3). Overall, 84 (98.8%) patients had an ‘improved’ or a better grade of GAIS at 0, 4, and 12 weeks, and 79 (92.9%) patients had an ‘improved’ or a better grade at 24 weeks (Table 2).

### 3.3. Safety

Adverse events included two cases (2.4%) of graft displacement, one case (1.2%) of postoperative fever, and one (1.2%) case of excessive bone regeneration, each of which was resolved appropriately. No other potential treatment-related adverse events were reported during the 24-week study period.

### 3.4. Satisfaction and Its Prediction Model

According to the questionnaires completed at the week 24 follow-up visit, the majority of patients 64 (75.3%) were satisfied or better. Patients’ main complaints about the outcome of the operation include insufficient improvement in concavity (18, 21.2%), foreign body sensation (3, 3.5%), and visual asymmetry (2, 2.4%). Subgroup analysis showed that foreign body sensation and the improvement in concavity were significantly associated with the overall satisfaction of patients (*p* = 0.017 and *p* < 0.001, respectively), while visual symmetry was not (*p* = 0.095) (Table 3).

Univariate analysis showed no significant correlation between satisfaction and the following factors: gender, age, BMI, ABO blood type, personality, main purpose, systolic blood pressure, diastolic blood pressure, blood glucose, heart rate, operative time, amount of Bio-Oss, number of titanium screws, size of Bio-Gide, simultaneous orthodontic treatment. Conversely, a significant correlation was shown between satisfaction and the following factors: FACE-Q score of expectations (*p* < 0.001), motivation (proactive vs. passive: 68.8% vs. 31.3%, *p* = 0.012), and history of periodontal disease (yes vs. no, 17.2% vs. 82.8%, *p* = 0.001) (Table 4). The potential influencing factors for satisfaction screened out in the univariate analysis were incorporated into the logistic regression analysis. The results show that the FACE-Q score of expectations, daily follow-up for 3 consecutive days, motivation, and history of periodontal disease were independent predictive factors of satisfaction (Table 5).

**Figure 4:**
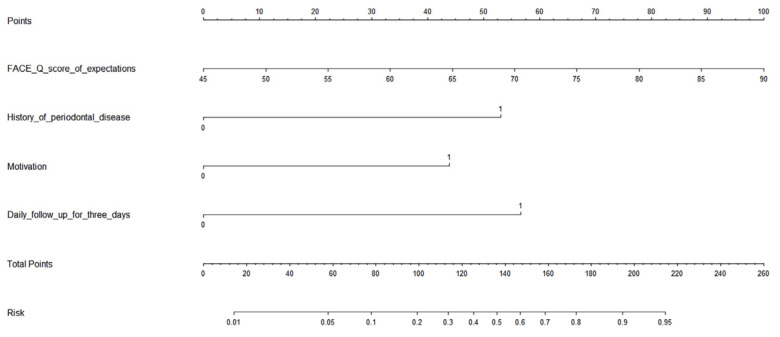
Nomogram (with the regression modeling strategies program) for predicting patient satisfaction after midface augmentation using Bio-Oss bone powder.

A nomogram model to predict the risk of dissatisfaction was constructed based on significant factors, as displayed in Figure 4. Factors in the nomogram model included the FACE-Q score of expectations, daily follow-up for 3 consecutive days or not, motivation, and history of periodontal disease. The C-index of this nomogram to assess prediction accuracy was 0.843 (*p* < 0.001), indicating good prediction performance. For example, assuming a patient with a FACE-Q score of 59 (32 points), a passive motivation, which means the patient was urged to seek this procedure (44 points), no history of periodontal disease (0 points), and no daily follow-up prescribed (57 points). Then, the total score is 133, and the probability of being dissatisfied with the result is estimated to be 46%.

### 3.5. Typical Cases

Case 1, female, 26 years old, was diagnosed with midface depression and bilateral nasolabial folds. The preoperative WSRS score was 3. The filling amount of Bio-Oss bone powder was 1 g on each side. Postoperative follow-up visits were performed according to the study design. At week 24, the evaluator results showed a WSRS score of 2 and a GAIS classification of ‘very much improved’. The patient expressed great satisfaction (Supplementary Figure S1). Three-dimensional reconstructed CT images of the facial skeleton show the contour and thickness of the augmentation area before and after the operation (Supplementary Figure S2).

Case 2, female, 25 years old, was diagnosed with midface depression and bilateral nasolabial folds. The preoperative WSRS score was 2. The filling amount of Bio-Oss bone powder is 1.5 g on the left side and 1 g on the right side. At week 24, the evaluator results showed a WSRS score of 1 and a GAIS classification of ‘much improved’. The patient expressed great satisfaction (Supplementary Figure S3).

## 4. Discussion

The basis of every attractive face is a skeleton that is proportional and well-balanced. The aesthetic outcome is also ultimately determined by the facial soft tissue being well supported by a skeletal foundation [23]. A common skeletal defect of human appearance is midface retrusion, which is usually centered in the middle of the maxilla. The localized retrusion may be congenital, developmental, or acquired [24]. Some normal-looking people still manifest slight retrusion defects in the upper segment of the nasolabial folds due to a mild depression of bone tissue around the pyriform aperture, although the condition is not prominent [18]. Nasolabial folds are the folds on both sides of the face, starting from the outer corners of the nose and ending at the corners of the mouth. The nasolabial folds stemming from such simple bone retrusion are common in young individuals and are mainly manifested as a broad concavity in the upper segment of the nasolabial fold, as well as flat skin on both sides of the nose [18]. However, in middle-aged and older populations, this condition often coexists with other subtypes of nasolabial folds. Nowadays, there is an increasing demand for aesthetic correction of them.

Midface augmentation is a procedure that can produce a noticeable aesthetic improvement in the above defects by altering the nasolabial angle, the projection of the alar base, and the vertical plane of the lip [25]. Many materials have been used for midface augmentation. Generally, these materials are of either biological or artificial origin. The former can be divided into autologous, allogenic, or xenogenic implants [26]. Autologous tissue implants have inherent disadvantages, such as unpredictable resorption and donor site morbidity. Most of the other implants do not have these disadvantages, but need to meet certain requirements, including biocompatibility, shape maintenance, ease of use, ease of manipulation, cost-effectiveness, etc.

Bio-Oss bone powder is one of the synthetic biocompatible materials, and more closely resembles the authentic hydroxyapatite in bone. The structure of Bio-Oss bone powder consists of a wide, interconnecting pore system that enables it to serve as a physical scaffold for osteogenic cells, thus promoting the migration and attachment of these cells [27]. In addition, Bio-Oss is able to upregulate some functional activities of osteoblast-like cells: signal transduction, cell cycle regulation, immunity, apoptosis, and vesicular transport [28]. Several experimental and clinical studies have demonstrated the osteoconductive potential of Bio-Oss [29]. This biomaterial has several advantages compared to the currently popular autogenous tissue implants, such as unlimited availability, permanent improvement, reduced morbidity of the donor site, and high clinical success rate [30]. In addition, the granular form provides surgical flexibility, allowing for small-volume augmentation when needed, and minimal operative exposure and tissue dissection. The Bio-Gide membrane is a newly developed absorbable collagen membrane. The ability of Bio-Gide to promote progenitor cell chemotaxis and adhesion, along with its intraoperative maneuverability, space maintenance capacity, low immunogenicity, and physiological degradation, makes it an ideal material for barrier membranes [31]. The joint application of Bio-Oss and Bio-Gide is based on the concept of shielding guided bone regeneration areas with membranes for preventing the rapid growth of epithelial cells or connective tissue cells, thereby promoting the undisturbed regeneration of bone. When the collagen membrane was anchored with screws and the area filled with Bio-Oss was protected, the guided new bone formation was significant. From our point of view, this approach of directly addressing the defects is preferable to techniques that attempt to camouflage them, such as tissue redraping alone or the use of supraperiosteal soft tissue fillers. What is more, this approach offers a permanent aesthetic improvement. Therefore, we recommend the use of Bio-Oss and Bio-Gide for midface augmentation to improve midface retrusion and associated nasolabial folds.

Although based on a simple subjective assessment, the WSRS is able to provide a valid and reproducible grading system of the nasolabial folds, thereby allowing the plastic surgeon the opportunity to evaluate treatment outcomes in quantitative terms. Another further advantage of the WSRS, for clinical purposes, is that each grade on the scale represents a clinically meaningful change in nasolabial fold severity from the adjacent grades [19]. The WSRS evaluation of this study, conducted by blinded evaluators, indicates that approximately half of the patients had at least a 1-grade decrease at 24 weeks from baseline. There are two reasons why the percentage of patients with significant grade improvement is not as high. On the one hand, the majority of patients in this study were young and had less severe nasolabial folds on average, with 80 (94.1%) patients having WSRS grades concentrated in grade 2 (mild) and grade 3 (moderate). On the other hand, the investigators scored strictly according to the WSRS evaluation criteria, and did not exaggerate the changes in the nasolabial folds. Therefore, despite the presence of visual improvement, some nasolabial folds were still evaluated as the same grade after the surgery because they did not achieve a 1-grade change. The mean WSRS score decreased from 2.56 ± 0.61 at baseline to 1.45 ± 0.50, 1.99 ± 0.33, and 2.05 ± 0.30 at 4, 12, and 24 weeks, respectively, with a highly statistically significant difference at each stage (*p* < 0.001). The average WSRS score improvements from baseline were 1.31 ± 0.56, 1.12 ± 0.63, 0.58 ± 0.62, and 0.52 ± 0.57 at 0, 4, 12, and 24 weeks, respectively, indicating that this technique produced significant aesthetic improvements. Notably, despite the guided bone regeneration, the mean vertical height of the augmentation at week 24 was slightly less than at weeks 4 and 12, suggesting that the rate of resorption and degradation of Bio-Oss may be slightly faster than the rate of guided generation of new bone in this application. Therefore, our future aim is to anticipate this phenomenon when designing the surgery, and to apply moderate overfilling during the procedure.

A clinically successful and meaningful outcome was defined as an ‘improved’ or a better GAIS rating. In total, 79 out of 85 patients were determined to be improved at 24 weeks using the GAIS. Therefore, 92.9% of patients met the primary efficacy endpoint. Furthermore, 98.8% of patients were determined to be clinically meaningful (at least 1 grade improvement) at 4 and 12 weeks. In conclusion, the results of the GAIS and WSRS scores confirm the satisfactory efficacy of this technique.

Four cases (4.7%) of adverse events were observed during the study period. Two patients had graft displacement after the surgery due to compression and impact after dropping a cell phone, and each underwent a second surgery for correction after 3 months of observation. One patient underwent an additional surgery of bone grinding for postoperative excessive bone regeneration. Another patient developed a persistent fever of >38.5 °C on the second day after the procedure, and symptoms resolved completely after empiric treatment with oral antibiotics for 7 days. Accordingly, for this technique, we recommend that additional postoperative instructions be given to the patient to avoid squeeze or accidental impact on the surgical area.

Patient satisfaction is of utmost importance to aesthetic plastic surgeons, as it helps to determine the quality of their practice and to continuously improve it. However, compared with surgeons, patients tend to be more subjective and more hypercritical about the details, which may be the reason for the different satisfaction rates in this study at week 24 between the patients and researchers (75.3 vs. 91.8%). According to the results of the patient self-evaluation at week 24, 67 (78.8%) patients expressed a self-feeling of being more attractive and a willingness to recommend this treatment to others.

Subgroup analysis showed that foreign body sensation and the improvement in concavity were significantly associated with the overall satisfaction of patients, while visual symmetry was not, indicating that plastic surgeons should pay more attention to the improvement in concavity during augmentation. However, a possible reason that the present study did not identify the relationship between visual symmetry and overall satisfaction was the low statistical power resulting from the low rate of visual asymmetry and overall dissatisfaction.

In order to further improve patient satisfaction in the future, we then analyzed the factors related to satisfaction. We believe that the identification of preoperative influencing factors alone cannot accurately predict postoperative patient satisfaction. Therefore, we screened various factors before and after the surgery. As a result, univariate and multivariate logistic regression analysis showed that the FACE-Q score of expectations, daily follow-up for 3 consecutive days, motivation, and history of periodontal disease were independent influencing factors of satisfaction after this procedure.

In this study, 22 patients had a history of periodontal disease (satisfaction rate, 50.0%), and 63 patients had no history of periodontal disease (satisfaction rate, 84.1%). Univariate analysis revealed that the difference was significant (*p* = 0.001), suggesting that patients with a history of periodontal disease are at a higher risk of dissatisfaction after the surgery than patients without. Multivariate logistic regression analysis subsequently showed that the difference was significant (*p* = 0.023), which indicates that a history of periodontal disease is a negative predictor of satisfaction after the surgery. The OR (7.361; 95% CI, 1.316–41.186) indicate that patients with a history of periodontal disease have a 7.361-fold higher risk of dissatisfaction than those without. The mechanism of the association between the history of periodontal disease and postoperative satisfaction is not fully understood. We assume that patients with periodontal disease tend to have a relatively poor periodontal tissue status, which can negatively affect bone regeneration and incision healing. In addition, periodontal disease is often associated with long-term, poor oral care habits, which are also detrimental to the results.

A total of 52 patients had a proactive motivation (satisfaction rate: 84.6%), and 33 patients had a passive motivation (satisfaction rate: 60.6%). Univariate analysis showed a significant difference (*p* = 0.012), indicating that patients with passive motivation are at a higher risk of dissatisfaction than patients with proactive motivation. In addition, multivariate logistic regression analysis revealed that the difference was significant (*p* = 0.014), which indicates that passive motivation is a negative predictor for satisfaction after the surgery. The OR (5.209; 95% CI, 1.402–19.356) reveals that patients with passive motivation have a 5.209 higher risk of dissatisfaction than those with proactive motivation. The patient’s motivation for surgery is an easily overlooked item in preoperative screening programs. However, our findings confirm that patient motivation can be involved in predicting outcomes. This correlation is easy to understand. A reluctant patient who is advised or urged by friends or family to seek aesthetic improvement naturally has a more pessimistic view of the outcome compared to a proactive patient. We accordingly recommend it as an important preoperative screening item for all cosmetic procedures.

In this work, we applied a FACE-Q of expectations preoperatively to help us quantify the patients’ expectations. Both univariate and multivariate analyses showed a significant correlation between FACE-Q and postoperative patient satisfaction: patients with higher scores (higher expectations) are at a higher risk of dissatisfaction than patients with lower scores. The OR (1.087; 95% CI, 1.015–1.165) shows that patients each with a 1-point increase are at a 1.087-fold higher risk of dissatisfaction. It is well known that patients with higher or more unrealistic expectations are unlikely to be fully satisfied with the outcomes. The FACE-Q allows us to classify expectations more finely, thus improving the accuracy of outcome prediction.

The data show that 46 patients practiced daily follow-up visits for 3 consecutive days as required (satisfaction rate, 82.6%), while 39 patients did not (satisfaction rate, 66.7%). Multivariate logistic regression analysis revealed that the difference was significant (*p* = 0.012), which shows that daily follow-up visits for 3 consecutive days were a protective and positive factor for postoperative satisfaction. The OR (8.420; 95% CI, 1.601–44.281) reveals that patients who practiced daily follow-up visits for 3 consecutive days had an 8.42-fold lower risk of dissatisfaction than those who did not. We provide possible explanations for the correlation. First of all, the daily follow-up visits for 3 consecutive days were an important part of our overall treatment procedure. During these 3 days, we would assist patients with drainage, adjust or remove the drainage strips, closely monitor the recovery, and treat accordingly. It is certainly difficult to achieve satisfactory results for patients who did not receive the complete treatment procedure. Secondly, in our experience, patients with poor compliance tend to be more hypercritical, which is also a possible reason.

It should be noted that all included variables had independent effects in the adjusted model, with no statistically significant interactions, indicating additive influences at the patient level. Accordingly, we proposed a nomogram with a high prediction rate cap (0.95) based on these factors. We believe that the exact causal mechanism of any associations observed in this study is a secondary consideration, and does not affect the predictive capacity of the nomogram. In conclusion, for this innovative procedure, identifying these influencing factors of patient satisfaction and applying a corresponding nomogram can help select patients, optimize perioperative management, and ultimately improve surgical outcomes.

There are several limitations. First, it is difficult to generalize our conclusions, given that the study was conducted at a single institution. Second, the number of patients in each group may weaken our statistical evaluation ability. The lack of a control group also limits the evaluation of potential benefits of midface augmentation with Bio-Oss bone powder. Third, the surgical outcomes and aesthetic evaluations were subjective reports from the patients and researchers, rather than objective indicators. Therefore, there may be potential information bias. Fourth, although photographs should be taken according to the same standard procedure, it is difficult to implement adequately and uniformly. Slight variations in photography angle and ambient light may also lead to potential deviations. Moreover, the amount of bone regeneration guided by Bio-Oss bone powder varies from person to person, and is not yet fully controllable. Therefore, there is still room for further exploration and improvement in the aesthetic improvement ability, predictability, and controllability of midface augmentation with Bio-Oss bone powder.

## 5. Conclusions

Midface augmentation using Bio-Oss bone powder and the Bio-Gide collagen membrane is a safe and effective tool for improving midface retrusion and the resulting nasolabial folds, and we advocate this method. The advantages also include the filling mode of guided autogenous bone regeneration, almost no foreign body sensation, and no impact on facial expression. Nevertheless, it should be noted that this method is not suitable for nasolabial folds other than the bone retrusion type. Foreign body sensation and the improvement in concavity may be significantly associated with overall patient satisfaction. The FACE-Q score of expectations, daily follow-up for 3 consecutive days, motivation, and history of periodontal disease were independent influencing factors of patient satisfaction. Accordingly, we developed a nomogram that can accurately predict the satisfaction rate after midface augmentation with Bio-Oss bone powder. This predictive model is easy to learn to help surgeons predict patient satisfaction and optimize perioperative clinical decision making. With the aim of becoming widely used and achieving better outcomes, long-term prospective studies with a larger sample size for this new surgical technique should be conducted.

## Figures and Tables

**Table 1:** Comparison (with a *t*-test) of WSRS scores ^a^.

Follow-Up Duration	WSRS	*p* Value
Baseline	2.56 ± 0.61	
Week 0	1.26 ± 0.44	<0.001
Week 4	1.45 ± 0.50	<0.001
Week 12	1.99 ± 0.33	<0.001
Week 24	2.05 ± 0.30	<0.001

^a^ Values are expressed as mean ± SD. Abbreviations: WSRS = Wrinkle Severity Rating Scale.

**Table 2:** Masked evaluation of aesthetic results using GAIS ^a^.

Grade	*n* (%)			
	Week 0	Week 4	Week 12	Week 24
Very much improved	81 (95.3%)	78 (91.8%)	76 (89.4%)	54 (63.5%)
Much improved	4 (4.7%)	6 (7.1%)	3 (3.5%)	23 (27.1%)
Improved	0	1 (1.2%)	5 (5.9%)	2 (2.4%)
No change	0	0	1 (1.2%)	4 (4.7%)
Worse	0	0	0	2 (2.4%)

^a^ Abbreviations: GAIS = Global Aesthetic Improvement Scale.

**Table 3:** Subgroup analysis (with a x^2^ test) of patient satisfaction.

	Overall Satisfied, *n* (%)			
	Yes	No	*x* ^2^	*p* Value
Insufficient improvement in concavity			64.555	<0.001
Yes	0	18		
No	64	3		
Visual asymmetry			2.785	0.095
Yes	0	2		
No	64	19		
Foreign body sensation			5.746	0.017
Yes	0	3		
No	64	18		

**Table 4:** Factors affecting patient satisfaction after midface augmentation (with *t*-test and x^2^ test) ^a^.

Variable	Satisfaction			
	Satisfied (*n* = 64)	Dissatisfied (*n* = 21)	*x* ^2^	*p* Value
Gender			0.672	0.412
Male	2 (3.1%)	0 (0.0%)		
Female	62 (96.9%)	21 (100.0%)		
Age (years)	27.3 ± 4.6	27.2 ± 3.1		0.956
BMI (kg/m^2^)	19.7 ± 1.5	19.4 ± 1.4		0.457
ABO blood type			1.440	0.696
A (+)	17 (26.6%)	4 (19.0%)		
AB (+)	11 (17.2%)	3 (14.3%)		
B (+)	16 (25.0%)	8 (38.1%)		
O (+)	20 (31.1%)	6 (28.6%)		
Personality			1.611	0.204
Extrovert	39 (60.9%)	16 (76.2%)		
Introverted	25 (39.1%)	5 (23.8%)		
Motivation			6.256	0.012
Proactive	44 (68.8%)	8 (38.1%)		
Passive	20 (31.3%)	13 (61.9%)		
Main Purpose			1.745	0.186
Midface depression	26 (40.6%)	12 (51.7%)		
Nasolabial folds	38 (59.4%)	9(42.9%)		
History of periodontal disease			10.209	0.001
Yes	11(17.2%)	11(52.4%)		
No	53(82.8%)	10(47.6%)		
FACE-Q score of expectations	59.0 ± 7.5	68.1 ± 11.1		<0.001
Systolic blood pressure (mmHg)	105.1 ± 12.1	100.9 ± 10.5		0.157
Diastolic blood pressure (mmHg)	73.2 ± 9.7	70.9 ± 9.0		0.327
Blood glucose (mmol/L)	6.4 ± 0.9	6.5 ± 0.8		0.595
Heart rate (bpm)	85.8 ± 5.8	83.7 ± 7.9		0.182
Operative time (min)	101.3 ± 10.4	101.8 ± 10.5		0.842
Amount of Bio-Oss (g)	2.2 ± 0.5	2.3 ± 0.4		0.632
Number of titanium screws	11.0 ± 2.5	11.3 ± 1.7		0.554
Size of Bio-Gide			0.005	0.943
25 × 25 mm	28 (43.8%)	9 (42.9%)		
30 × 40 mm	36 (56.3%)	12 (57.1%)		
Simultaneous orthodontic treatment			0.008	0.930
Yes	22 (34.4%)	7 (33.3%)		
No	42 (65.6%)	14 (66.7%)		
Daily follow-up for 3 days			2.884	0.089
Yes	38 (59.4%)	8 (38.1%)		
No	26 (40.6%)	13 (61.9%)		

^a^ Values are expressed as *n* (%) or mean ± SD.

**Table 5:** Logistic regression of predictors of patient satisfaction after midface augmentation.

Variable	B	SE	Wals	*p* Value	OR	95% CI
FACE-Q score of expectations	0.084	0.035	5.631	0.018	1.087	1.015–1.165
History of periodontal disease	1.996	0.879	5.163	0.023	7.361	1.316–41.186
Motivation	1.650	0.670	6.075	0.014	5.209	1.402–19.356
Daily follow-up for 3 days	2.131	0.847	6.328	0.012	8.420	1.601–44.281

## Data Availability

Not applicable.

## Supplementary Materials

The following supporting information can be downloaded at: https://www.mdpi.com/article/10.3390/jcm12030959/s1, Table S1: Nasolabial Folds Assessment according to Wrinkle Severity Rating Scale before Midface Augmentation. Table S2: Results of Patient Self-Evaluation at Week 24.
