# Supplementary material for: Safety and Efficacy of Midface Augmentation Using Bio-Oss Bone Powder and Bio-Gide Collagen Membrane in Asians
Source: J Clin Med. 2023 Jan 26;12(3):959. doi: 10.3390/jcm12030959 (PMC9917653; doi:10.3390/jcm12030959)
Supplement: Supplementary file 1 [file jcm-12-00959-s001.zip › jcm-2182785-supplementary.pdf]

Supplementary Table S1. Nasolabial Folds Assessment according to Wrinkle Severity Rating Scale before Midface Augmentation

Grade	Description	N	%
1 (absent)	No visible fold; continuous skin line.	1	1.2
2 (mild)	Shallow but visible fold with a slight indentation; minor facial feature.	39	45.9
3 (moderate)	Moderately deep folds; clear facial features visible at normal appearance but not when stretched.	41	48.2
4 (severe)	Very long and deep folds, prominent facial features; Less than 2 mm visible fold when stretched.	4	4.7
5 (extreme)	Extremely deep and long folds, detrimental to facial appearance; 2–4 mm visible V-shaped fold when stretched.	0	0

Supplementary Table S2. Results of Patient Self-Evaluation at Week 24

		N	%
Overall satisfied	Yes	64	75.3
	No	21	24.7
Feel more attractive	Yes	67	78.8
	No	18	21.2
Willingness to recommend to	Yes	67	78.8

others	No	18	21.2

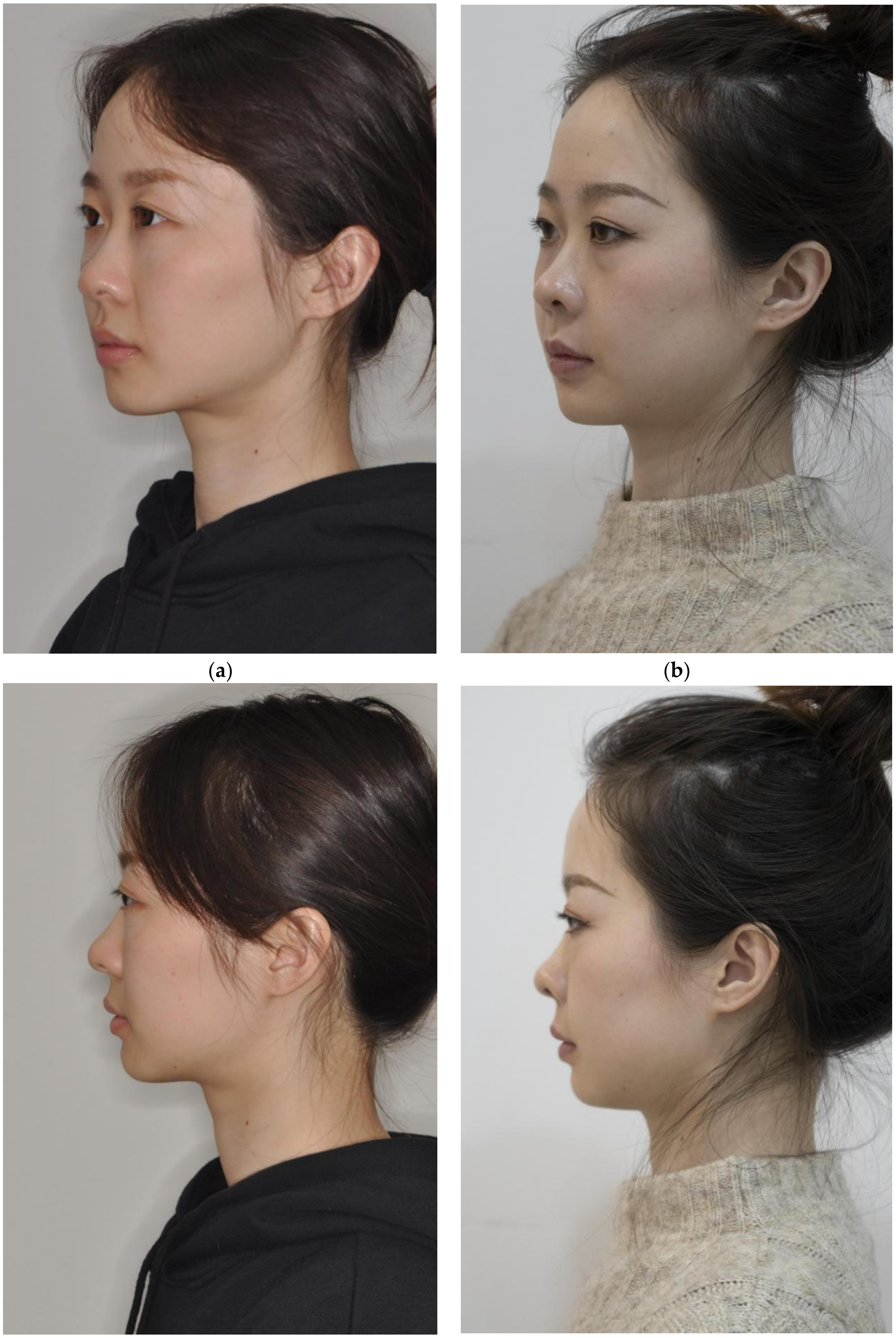

(**a**) (**b**) (**c**) (**d**)

Figure S1. (**a**) Preoperative oblique view of patient 1; (**b**) 24 weeks postoperative oblique view of patient 1; (**c**) preoperative lateral view of patient 1; (**d**) 24 weeks postoperative lateral view of patient 1.

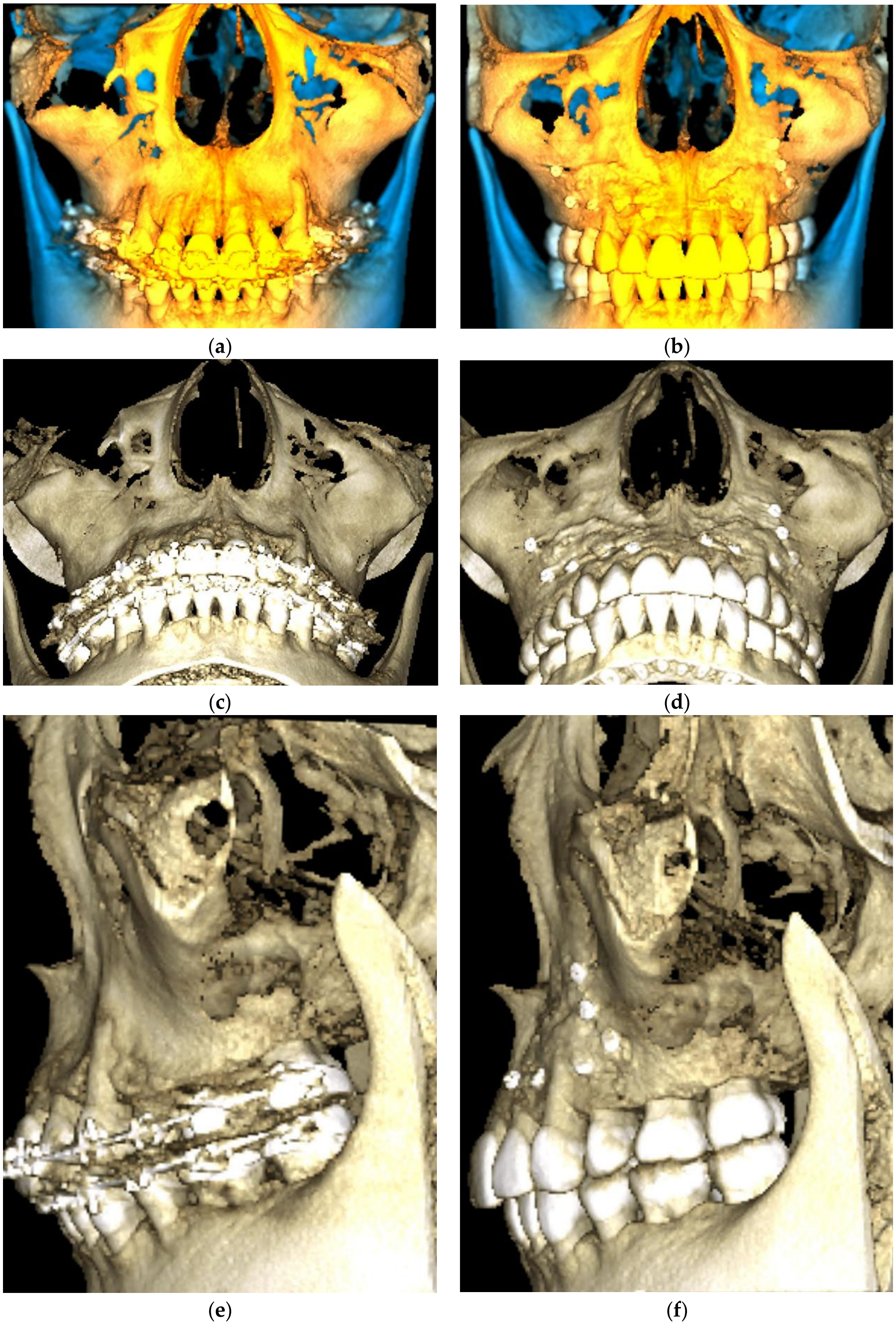

Figure S2. (**a**) Front view of preoperative three-dimensional reconstructed CT image of patient 1; (**b**) front view of 24 weeks

postoperative three-dimensional reconstructed CT image of patient 1; (**c**) bottom view of preoperative three-dimensional reconstructed CT image of patient 1; (**d**) bottom view of 24 weeks postoperative three-dimensional reconstructed CT image of patient 1; (**e**) lateral view of preoperative three-dimensional reconstructed CT image of patient 1; (**f**) lateral view of 24 weeks postoperative three-dimensional reconstructed CT image of patient 1.

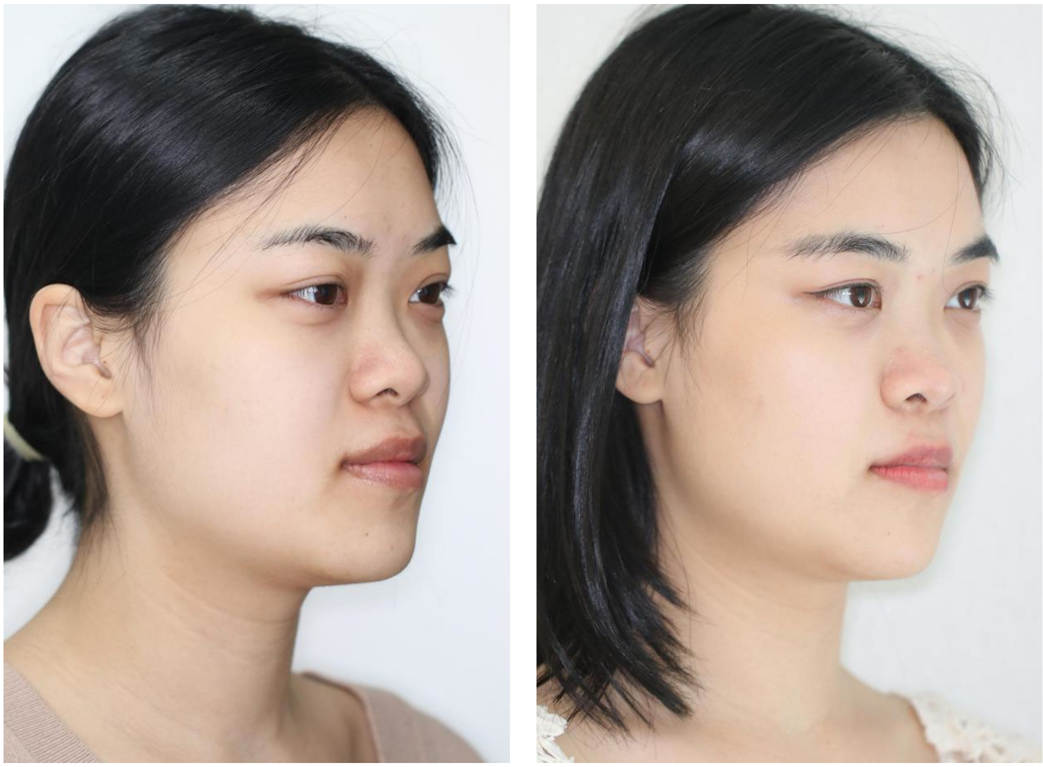

(**a**) (**b**)

Figure S3. (**a**) Preoperative oblique view of patient 2; (**b**) 24 weeks postoperative oblique view of patient 2.
